# Activated networking of platelet activating factor receptor and FAK/STAT1 induces malignant potential in BRCA1-mutant at-risk ovarian epithelium

**DOI:** 10.1186/1477-7827-8-74

**Published:** 2010-06-24

**Authors:** Lifang Zhang, Dan Wang, Wei Jiang, Dale Edwards, Weiliang Qiu, Lisa M Barroilhet, Jung-hyun Rho, Lianjin Jin, Vanitha Seethappan, Allison Vitonis, Jianliu Wang, Samuel C Mok, Christopher Crum, Daniel W Cramer, Bin Ye

**Affiliations:** 1 Department of Obstetrics and Gynecology, Brigham and Women's Hospital, Boston, MA, USA; 2 Channing Laboratory, Brigham and Women's Hospital, Boston, MA, USA; 3 Obstetrics and Gynecology Department, Peking University People's Hospital, Beijing, China; 4 Obstetrics and Gynecology Hospital of Fudan University, 419 Fang Xie Road, Shanghai 200011, China; 5 Department of Gynecologic Oncology, University of Texas M. D. Anderson Cancer Center, Houston, TX, USA; 6 Department Pathology, Brigham and Women's Hospital, Boston, MA, USA

## Abstract

**Objectives:**

It is essential to understand the molecular basis of ovarian cancer etiology and tumor development to provide more effective preventive and therapeutic approaches to reduce mortality. Particularly, the molecular targets and pathways involved in early malignant transformation are still not clear. Pro-inflammatory lipids and pathways have been reported to play significant roles in ovarian cancer progression and metastasis. The major objective of this study was to explore and determine whether platelet activating factor (PAF) and receptor associated networking pathways might significantly induce malignant potential in BRCA1-mutant at-risk epithelial cells.

**Methods:**

BRCA1-mutant ovarian epithelial cell lines including (HOSE-636, HOSE-642), BRCA1-mutant ovarian cancer cell (UWB1.289), wild type normal ovarian epithelial cell (HOSE-E6E7) and cancerous cell line (OVCA429), and the non-malignant BRCA1-mutant distal fallopian tube (fimbria) tissue specimens were used in this study. Mutation analysis, kinase microarray, western blot, immune staining, co-immune precipitation, cell cycle, apoptosis, proliferation and bioinformatic pathway analysis were applied.

**Results:**

We found that PAF, as a potent pro-inflammatory mediator, induced significant anti-apoptotic effect in *BRCA1-*mutant ovarian surface epithelial cells, but not in wild type HOSE cells. With kinase microarray technology and the specific immune approaches, we found that phosphor-STAT1 was activated by 100 nM PAF treatment only in BRCA1-mutant associated at-risk ovarian epithelial cells and ovarian cancer cells, but not in BRCA1-wild type normal (HOSE-E6E7) or malignant (OVCA429) ovarian epithelial cells. Co-immune precipitation revealed that elevated PAFR expression is associated with protein-protein interactions of PAFR-FAK and FAK-STAT1 in BRCA1-mutant ovarian epithelial cells, but not in the wild-type control cells.

**Conclusion:**

Previous studies showed that potent inflammatory lipid mediators such as PAF and its receptor (PAFR) significantly contribute to cancer progression and metastasis. Our findings suggest that these potent inflammatory lipids and receptor pathways are significantly involved in the early malignant transformation through PAFR-FAK-STAT1 networking and to block apoptosis pathway in BRCA1 dysfunctional at-risk ovarian epithelium.

## Background

The combination of mutation and aberrant expression of tumor suppressor genes is critical in cancer susceptibility and tumor progression. BRCA1 protein plays multiple essential functions such as tumor suppressor, transcriptional regulation and DNA repair in normal epithelial cells and stem cells [1]. An inherited *BRCA1*-mutation confers an increased risk of ovarian cancer, with lifetime risk estimates ranging from 10-60%, compared to a risk of less than 2% for the general population [2-4]. About 10% of women presenting with ovarian cancer carry a BRCA-mutation. Previous publications indicate that a *BRCA1 *mutation is associated with cancer progression through pathways of cell proliferation [5], differentiation [6], and apoptosis [7]. It is known that loss of BRCA1 function may ultimately activate JAK-STAT pathways and stimulate cell proliferation in breast, prostate, lung and ovarian cancer [8,9]. But it is still unclear what molecular mechanisms and targets characterize the early molecular events of malignant transformation.

Chronic inflammatory microenvironments have been hypothesized as the major factors predisposing ovarian [10,11] and other cancers [12]. Lipid mediators such as lysophosphatidic acid (LPA) and prostaglandin with their associated receptors and pathways such as COX have been shown to play a critical role in cancer initiation and progression [13,14]. Unfortunately, platelet activating factor (PAF, 1-O-alkyl-2-acetyl-sn-glycero-3-phosphorylcholine), as one of the most potent lipid mediators, has not yet been well studied in the regulation of early events of cancer transformation and progression [15], particularly with the at-risk *in vitro *and *in vivo *models. PAFR belongs to the G protein-coupled receptor (GPCR) protein family, and transduces cell signals via the G proteins and associated protein phosphorylation cascades [16,17]. When cells are exposed to PAF, it induces cell proliferation, activates tyrosine kinase [18] and protein phosphorylation [19] in human epithelial cells, skin fibroblasts [20], endothelial cells [21], lung fibroblasts cells [22], pulmonary vascular smooth muscle cells [23] and keratinocytes [24]. PAF plays significant roles in many biological pathways in inflammatory diseases and cancer progression [25,15,26]. Upon PAF/PAFR activation, the Signal Transducers and Activators of Transcription (STAT) pathways are activated by phosphorylation changes, dimerization, and translocated into the nucleus to activate transcription of specific genes in regulation of cellular functions [18,27]. Our earlier study demonstrated that platelet activating factor (PAF) and PAFR play a significant role in ovarian cancer progression and invasion through activation of a set of tyrosine phosphor-EGFR/Src/FAK/Paxillin[15]. In this study, we investigate the possibility that inflammation associated lipid mediator PAF might mediate the early BRCA-carcinogenic events using an *in vitro *at-risk model employing cancerous and non-cancerous ovarian *BRCA1*-mutant epithelial cells with over expression of PAFR.

## Methods

### Chemical reagents

DMSO, PAF, and ginkgolide B (>90% high-performance liquid chromatography grade), cell culture mediums of MCDB-105 and medium 199 were obtained from Sigma-Aldrich (St. Louis, MO). MEGM mammary epithelial cell growth medium was purchased from Lonza (Walkersville, MD) and RPMI 1640 from Invitrogen (Carlsbad, CA). CV-3988 (a selective inhibitor of PAFR) was purchased from Biomol International L.P (Plymouth Meeting, PA). Rabbit polyclonal antibody against PAFR was purchased from Cayman chemical company (Ann Arbor, Michigan). Phospho-kinase array kit and polyclonal antibody against phosphor-STAT1 was purchased from R&D (Minneapolis, MN). Rabbit polyclonal antibody against STAT1 and monoclonal antibody against FAK were purchased from Cell Signaling Technology (Boston, MA) and Invitrogen-Biosource International Inc. (Carlsbad, CA), respectively.

Immortalized *BRCA1*-mutant human ovarian surface epithelial cells (HOSE-636, and HOSE-642) were generated from women who underwent prophylactic oophorectomies because of predisposing *BRCA1 *mutation. Immortalized normal human ovarian surface epithelial cells (HOSE-E6E7 and HOSE-27) were derived from primary cultured cells of the fresh ovarian scrapings at the time of surgery for benign ovarian conditions without *BRCA1 *mutation. The UWB1.289 (here after referred to as UWB1) cell line is a serous-type ovarian cancer with a *BRCA1 *mutation (ATCC American Type Culture Collection, Manassas, VA) and OVCA429 cell line is a serous ovarian cancer without a *BRCA1 *mutation.

### Cell lines and cell culture

HOSE-E6E7, HOSE-27, HOSE-636, HOSE-642, and OVCA429 were cultured in M199/MCDB-105 with 15% fetal bovine serum (Gemini Bioproducts, West Sacramento, CA) and 1% antibiotic (200 mmol/L L-glutamine, 10,000 units penicillin, and 10 mg/mL streptomycin). UWB1 cells were cultured in MEGM/RPMI with 3% fetal bovine serum and 200 μg/ml Geneticin (GIBCO-Invitrogen, Carlsbad, CA). Cell culture conditions were maintained at 37°C under 5% CO_2 _and 95% air in a high-humidity chamber. All non-commercial cell lines were constructed from material collected under IRB approved protocols for collection of "de-identified" human subjects.

### BRCA1 mutation characterization analysis

BRCA1 exon sequence analysis: Genomic DNA was isolated from HOSE-636 cells with the Puregene Trial kit (Gentra Systems, Inc.), following the manufacturer's instructions. DNA was precipitated using a standard protocol using saturated NaCl and isopropanol. The DNA pellet was washed with 75% ethanol, air dried, and resuspended in Tris-EDTA (50 mM, pH6.8). Direct DNA sequencing for *BRCA1 *was done for all coding exons using Big Dye Terminator chemistry and an automated 3100 DNA sequencer (Applied Biosystems, Foster City, CA). In addition, HOSE-636, HOSE-642 and UWB1 cells with *BRCA1 *mutation were certified having the BRCA1 protein truncation by Western Blot according to the published method [28].

### Western blot

The cultured cells were washed twice with 1 × PBS and lysed with ice-cold cell lysis buffer (Cell Signaling Technology, Boston, MA). Protease inhibitors including phenylmethylsulfonyl fluoride (1 mM) and Protease Inhibitor Cocktail I and II (Sigma-Aldrich, St. Louis, Mo) were freshly added to the cell lysis buffer. After a brief vortexing and centrifuging at 4°C, the cell lysates were transferred to a new tube. The concentration of protein was determined with the Bradford Protein Assay Kit (Bio-Rad Laboratories). Protein lysates were subjected to 8-16% SDS-PAGE gel separation followed by transferring onto polyvinylidene difluoride membrane (Perkin-Elmer) using the Wet Transfer Cell (Invitrogen, Carlsbad, CA) and blocked with 5% fat-free dry milk at room temperature for 1 hour. After washing four times, membranes were incubated with primary antibody (Anti-BRCA1 antibody, Cell Signal Technology, Boston) in blocking solution (1:1000 dilution) at 4°C for overnight. Secondary antibody (1:1000) tagged with HRP was used to reveal the protein expression signals with chemiluminescent substrate kit (Pierce Chemical, Co.). Similar western blot protocol has been used for other protein detection including PAFR, STAT1 and FAK.

### Immune staining of PAFR in human tissue

Immunohistochemical analysis was performed for detecting PAFR protein expression in sixteen wild type and eight *BRCA1*-mutant patients using the EnVision/AP system (DakoCytomation) which used an anti-rabbit immunoglobulin conjugated to an alkaline phosphatase polymer (Labeled polymer-AP) with a red stain as positive after the addition of the Substrate-Chromogen solution. Slides were washed with deionized water and counterstained with hematoxylin and 5% ammonium hydroxide, and mounted in Accergel (Accurate Chemical and Scientific Corp). The image was recorded by a digital camera (Optronic Inc.). Human pancreatic tissue sections were used as a positive control.

### Cell treatment and proliferation assay

Cell proliferation was assessed by using a 3-(4,5-dimethylthiazol-2-yl)-2,5-diphenyltetrazolium bromide (MTT) assay (Promega, Madison, WI). Monolayer cells at 60-80% confluence were enzymatically removed using trpysin/EDTA and plated in 96-well flat-bottomed plates at a concentration of 1 × 10^5 ^per well. After overnight plating and starving for 24 h in FBS free medium, HOSE-E6E7, HOSE-27, HOSE-636, HOSE-642, and UWB1 cells were treated with different concentrations of PAF in 0.5% FBS medium daily for 3 days, raging from 0.1 nM to 100 nM. For details of PAF treatment and MTT assay, see our previous publication [15].

### Cell cycle and apoptosis analysis

Immortalized human ovarian surface epithelial cells HOSE-E6E7and HOSE-642 were rendered at confluence by incubation in M199/MCDB105 medium with FBS free overnight. After treatment with 100 nM PAF 24 and 72 h, cells were harvested and fixed with 70% cold ethanol in PBS buffer by suspending the cell pellet and incubating at -20°C within 7 days. The cells were resuspended in PI master mix (PI:40 ug/ml and RNase:100 ug/ml in PBS buffer without Ca^2+ ^and Mg^2+^) at a final cell density of 0.5 × 10^6 ^cells/ml. After transferring to a Falcon 2054 (Fisher# 149592A) tube and incubation at 37°C for 30 minutes, cells were subjected to analysis using a FACS Calibur™ Flow Cytometer (Becton-Dickson, San Jose, CA). Each experiment was repeated three times and the cell cycle profiles, apoptosis index were analyzed by ModFit LT software (Verity Software House, Inc., Topsham, ME) [29].

### Kinase microarray analysis

UWB1 cells were incubated in fresh RPMI/MEGM FBS-free medium overnight at 37°C in a humidified chamber, which were pretreated with ginkgolide B (GB,100 μM, as specific PAFR antagonist) 3 h before PAF (100 nM) treatment. Cells were harvested at certain time points after treatment by twice washing with 1 × PBS, then lysed with ice-cold cell lysis buffer (Cell Signal Technology), including protease inhibitors.

Phospho-Kinase Array kit (R&D, Minneapolis, MN) was used to detect the relative levels of phosphorylation of 46 kinase phosphorylation sites. The human Phospho-Kinase Array is divided into A and B parts with different sets of protein kinase targets, to maximize sensitivity and minimize cross-reactivity. Capture and control antibodies were spotted in duplicate on nitrocellulose membranes (A and B). First, the membranes were incubated with 1 mL of blocking buffer in 8-Well Multi-dish for 1 h on a rocking platform. About 310 μg of fresh protein lysates were diluted in 1 ml array buffer 1 (1:5) and incubated (A and B membrane) overnight at 4°C. Membranes were then removed to the same container and washed 3 times using 1 × washing buffer. A detection antibody cocktail (for A and B membrane, respectively) diluted with 1 × array buffer was incubated for 2 h at room temperature. After an additional 3 washes, 30 min for each, streptavidin-HRP (1:2000) was used to reveal protein phosphorylation by chemiluminescent kit (Pierce Chemical, Co.) and quantified via an Axon Genepix scanner (Molecular Devices, Co.) and analyzed with ArrayVision software.

### Co-immunoprecipitation

A 400 μg protein lysate of HOSE-642 cells with PAF treatment were incubated with 5 μg antibodies of PAFR or phosphor-PAK with 400 μL immunoprecipitation buffer for 2 hours at 4°C. Protein A/G agarose beads were added for overnight incubation. After five times wash, the protein of interest was eluted by SDS/reducing sample buffer and boiling for 3 min and then subjected to 7.5% SDS-PAGE gel separation. The protein was transferred to PVDF membrane (Perkin-Elmer) using the wet transfer cell (Invitrogen, Carlsbad, CA) and blocked with 5% fat-free dry milk at room temperature for 1 hour. After washing four times, membranes were incubated with different primary antibodies phosphor-FAK (1:1000 dilution) for PAFR co-immuneprecipitation, and PAFR (1:1000 dilution) for FAK co-immuneprecipitation, respectively, at 4°C overnight. Following an additional three washes, protein expression signals were detected by HRP with chemiluminescent kit (Pierce Chemical, Co.) as described before. Protein IgG was used as control to confirm the immune specificity of protein-protein interaction and precipitation. A similar approach was used to define the protein-protein interaction between FAK and STAT1.

### Statistical analysis

The phosphor-kinase array data were analyzed by Origin 7.0 software. All other data were analyzed by using one-way factorial analysis of variance tests. Data collected from the treated and untreated cells with different dose or at different time point were analyzed by paired t-test and ANOVA test, respectively. The significance of protein expression on western blots and the immune staining intensity of PAFR in fallopian tube epithelial cells of patients with or without BRCA1+ mutation were analyzed by paired Student t-test.

## Results

### Characterization of BRCA1 mutation and over expression of PAFR in BRCA1 mutation ovarian epithelial cell lines and fallopian tube surface epithelial cells

**Figure 1:**
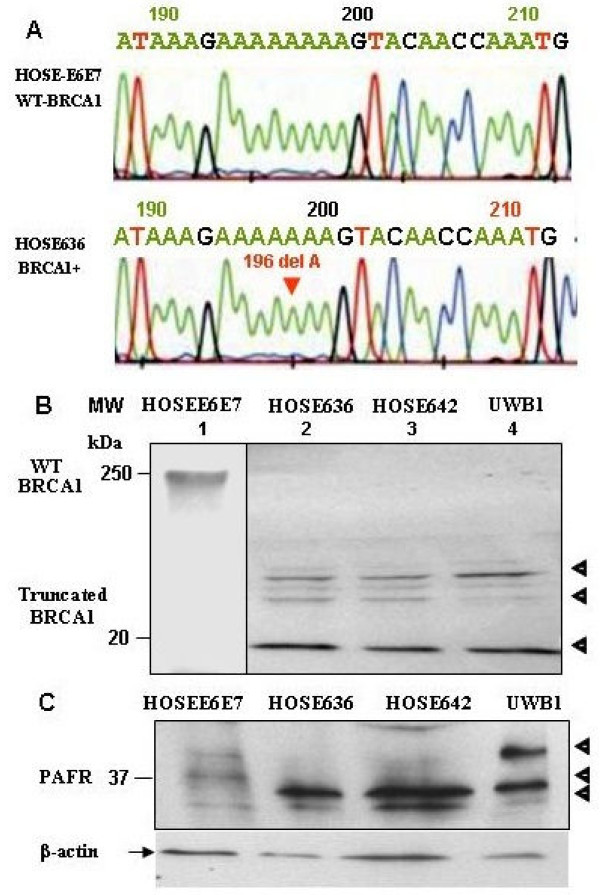
**Molecular characteristics of the BRCA1 mutation in (BRCA1^+^) ovarian epithelial cells**. **(A**) DNA extracted from the HOSE-636 and HOSE-E6E7 cells were subjected to BRCA1 gene sequence analysis with the standard protocol. 1961 delA mutation was detected in HOSE-636 cells. (**B**) Total protein lysis (30 μg) of Western blot showed pieces of mutant fragments of BRCA1 protein in HOSE-E6E7, HOSE-636 HOSE-642, and UWB1 cells were applied on SDS-PAGE and western blot analysis. Intact BRCA1 protein and its fragments were revealed by BRCA1 antibody. The separated western blots are required because of the nature and working conditions are different between BRCA1 intact protein and the truncated peptides. (**C**) PAFR protein was detected by western blot in wild type HOSE-E6E7 and BRCA1-mutant HOSE-636, HOSE-642, and UWB1 cell lines with a specific polyclonal antibody against human PAFR. The equal protein loading was normalized by β-actin.

DNA sequencing analysis of the germline DNA isolated from HOSE-636 cells revealed a mutation at 1961delA (with a stop codon at 700 of *BRCA1*) relative to the wild type *BRCA1 *sequence in HOSE-E6E7 cells (Fig. 1A). In addition, based on the method of [28], western blot analysis of the HOSE-636, HOSE-642 and UWB1 cells showed that the BRCA1-mutation cell lines consisted of BRCA1 truncated three peptide fragments of less than 25 kDa (see Fig. 1B lanes 2-4). The truncated BRCA1 protein peptides were not found in the wild-type normal ovarian epithelial cells (Fig. 1B) and ovarian cancer cells such as OVCA429 (data not shown).

**Figure 2:**
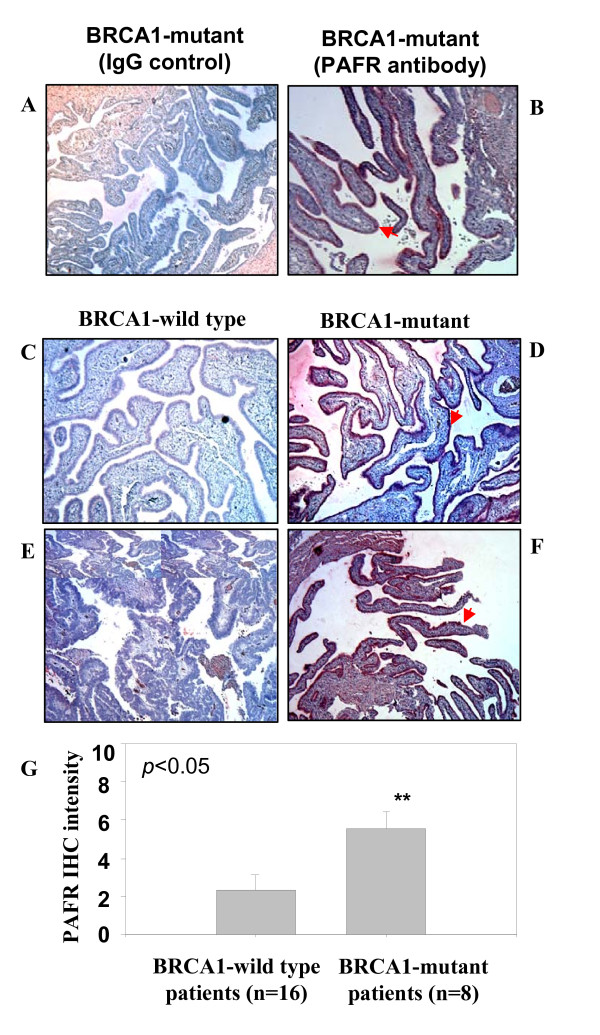
**Immune staining of PAFR expression in epithelial cells of fallopian tube fimbria of BRCA1^+ ^patients**. (**A, B**) Immune staining of epithelial cells of fallopian tube fimbria of BRCA1^+ ^patients with and without PAFR antibody. (**C, E**) Negative PAFR staining on the BRCA1 wild type epithelial cells of fallopian tube fimbria. (**D, F**) Positive immune staining of PAFR in epithelial cells of fallopian tube fimbria of the BRCA1^+ ^patients. (**G**) Summary of the intensity scale (mean value with standard error bar) of immune histochemistry staining of the epithelial cells of fallopian tube collected from the patients without (n = 16) and with BRCA1-muation (n = 8) (*p *< 0.05). The grading system (1-10) of the immune intensity was used for semi-quantification and same set of slides were read by two independent investigators.

Western blot analysis of PAFR protein expression revealed three protein bands corresponding to PAFR expression with slight variations between different cell lines. One PAFR protein band (upper) with a high molecular weight was strongly detected in UWB1 cells. The second band was specifically over-expressed in all *BRCA1-*mutant ovarian epithelial cell lines (HOSE-636, HOSE-642 and UWB1), but less expressed in wild-type normal HOSE-E6E7 cells. The lower molecular weight PAFR band was barely detectable, which may correspond to the constitutive level of PAFR expression in both wild type and *BRCA1*-mutant ovarian epithelial cells (Fig. 1C). This data was consistent with our previous finding that multiple isoforms of PAFR were expressed in human ovarian epithelial cancer cells and in other mammalian cells [15,30]. To further determine whether over expression of PAFR was associated with *BRCA1 *mutations, immunohistochemical staining (IHC) was performed on tubal fimbral tissue slides from women with inherited BRCA mutations and randomly selected BRCA1 wild type control fallopian tubes. Immunohistochemical staining for PAFR was positive in epithelial from the women with BRCA1 mutations (Fig. 2BDFG, n = 8), but less in the wild type controls (Fig. 2CEG, n = 16, *p *< 0.05).

### Lipid PAF activates PAFR/BRCA1/STAT networking pathway in BRCA1-mutant ovarian epithelial cancer cells

**Figure 3:**
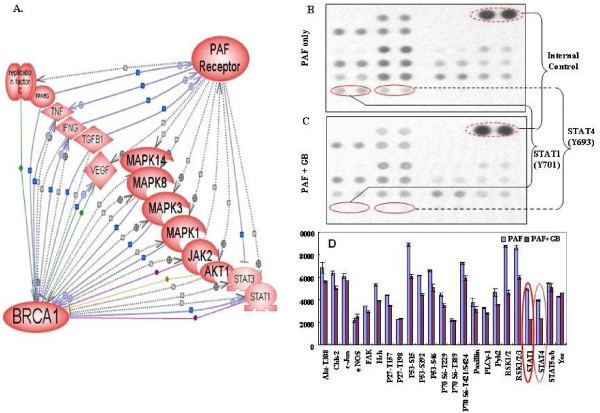
**Bioinformatic pathway and protein-protein interaction between PAFR-BRCA1**. (**A**). Pathway Studio software (Ariadne Inc.) and Human ResNet Mammalian Database were used in the study. Antibody microarray based protein kinase profiling and identification of STAT1 and STAT4 in BRCA1-mutant and PAFR-over expression (PAFR^+^) ovarian epithelial cancer cells. UWB1 cells were treated with PAF (100 nM) for 24 hours. Images of kinase arrays of signals after the incubation with 310 μg protein lysate of UWB1 cells treated with 100 nM PAF only (**B**) or with combination of 100 μM ginkgolide B (GB) and 100 nM PAF (**C**). The duplicated internal positive controls were used to calibrate the detection signal of membranes of the treated and untreated experiments. The summary of kinase expression profile of the UWB1 cells with different treatments and significance comparison by the paired student t-test (**D**).

Activated platelets and platelet activating factor (PAF) are often associated with inflammatory conditions and cancer [31,32]. Based on the previous publications, pathway bioinformatic software revealed that about 14 proteins are mediated in the functional regulations and protein-protein interactions between PAFR and BRCA1 and majority of interactive nodes are protein kinases (Fig. 3A). To investigate whether ovarian epithelial cells with *BRCA1*-mutation and PAFR-over-expression might be particularly sensitive and responsive to the inflammatory lipid mediators such as PAF, ovarian cancer cells (UWB1) with *BRCA1*-mutant and over expression of PAFR were treated with PAF (100 nM) for 24 hours, followed by protein phosphorylation detection and profiling using the kinase array. Among the 46 kinase phosphorylation sites/targets tested, we found that only one target (phosphor β-catenin) was significantly enhanced by PAF/PAFR blocking agent treatment at 24 h (not shown). Many phosphor-sites/proteins including STAT1, STAT3, STAT4, P53, AKT, TOR, P70 and RSK were significantly (*p *< 0.05) induced by PAF treatment (Fig. 3).

**Figure 4:**
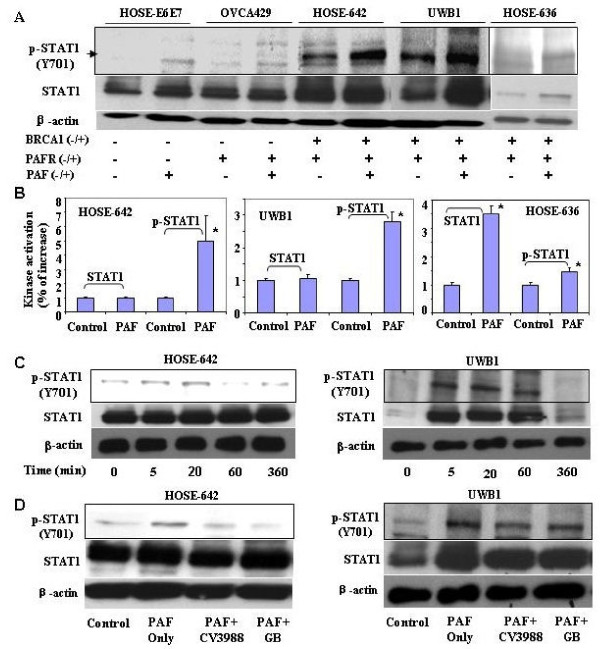
**Activated phospho-STAT1 in *BRCA1*^*+ *^ovarian epithelial cells**. (**A**) Wild type HOSE-E6E7 and OVCA429 cancer cells and *BRCA1*-mutant at-risk (HOSE-642, HOSE-636) cells and *BRCA1*-mutant ovarian cancer cells (UWB1) were subjected to PAF treatment for 20 minutes and phospho-STAT1 (Y701) detection by western blot. (**B**) Summary of intensity of SATA1 and phosphor-STAT1 induced by PAF treatment in HOSE-642, UWB1 and HOSE-636 cells. Phosphor-STAT1 was significantly (*p *< 0.01) increased in all BRCA1-mutant cells. **(C) **Activation of phosphor-STAT1 at different time points (0, 5, 20 60 min and 360 min) of PAF treatment (100 nM) in *BRCA1*-mutant HOSE-642 and UWB1 cells. (**D**) Equal amount of protein lysate of the cells of untreated, treated with PAF (100 nM) alone or in combination of PAF and PAFR-inhibitor (10 μM) and GB (100 μM) were applied in western blot detection. DMSO and pure IgG (not shown) were used as control treatment for 20 minutes in both HOSE-642 and UWB1 cells. Equal loading of protein was calibrated with expression of β-actin.

To further determine whether PAFR-STAT1 pathways might be involved in malignant transformation in *BRCA1*-mutant ovarian epithelial cells, wild-type ovarian epithelial normal cells (HOSE-E6E7) and ovarian cancer cells (OVCA429) were used as controls. *BRCA1*-mutant ovarian surface epithelial non-malignant (HOSE-642, HOSE-636) and malignant (UWB1) cells were treated with 100 nM PAF at different time points followed by phosphor-STAT1 detection using western blot with the specific phosphor-antibody (R&D, Minneapolis, MN). We found that the enhanced phosphor-STAT1 was neither observed in the wild type normal ovarian epithelial HOSE-E6E7 cells, nor in the wild type ovarian cancer cells (OVCA429), but only found in *BRCA1*-mutant cell lines (HOSE-642, HOSE-636, and UWB1) (Fig. 4A). In addition, we found that PAF induced a significant increase of both STAT1 and phosphor-STAT1 in HOSE-636 cells, but only increased phosphor-STAT1 in HOSE-642 and UWB1 cells (Fig. 4B). It appears that the increased phosphor-STAT1 peaked at 20 to 60 min of PAF treatment in *BRCA1*-mutant cell lines (e.g. HISE642 and UWB1) (Fig. 4C). To further investigate whether the activation of the phosphor-STAT1 pathway in *BRCA1*-mutant ovarian epithelial cells by PAF treatment is associated with PAFR, we examined the effect of various PAFR inhibitors such as CV3988 (10 μM), PAFR antagonist ginkgolide B (GB, 100 μM), and specific PAFR antibody (1:50, data not shown) on phosphor-STAT1 in both HOSE-642 and UWB1 cells at 20 min of treatment with PAF (100 nM). As expected, we found that pretreatment of CV3988, GB and PAFR antibody indeed showed significant inactivation of phosphor-STAT1 in BRCA1 mutation cell lines, particularly in HOSE642 cells (Fig. 4D).

### PAF induced cell proliferation, cell cycle and anti-apoptosis in BRCA1-mutant ovarian epithelial cells

**Figure 5:**
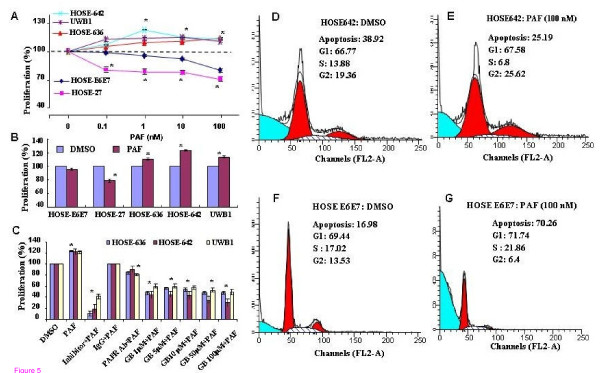
**PAF induced cell proliferation and anti-apoptosis in BRCA1-mutant ovarian epithelial cells**. (**A**) The induced cell proliferation pattern in wild type HOSE-E6E7 and HOSE-27 cells and *BRCA1-*mutant HOSE-642 and HOSE-636 and ovarian malignant cells (UWB1) was affected by 72 hours treatment with different concentrations of PAF. Significant increase or decrease of cell proliferation induced by PAF treatment was indicated by symbol star (* p < 0.05). (**B**) Differential cell proliferation pattern was affected by PAF (1 nM) treatment in wild type of HOSE-E6E7 and HOSE-27 cells and *BRCA1*-mutant non-malignant HOSE-642 and HOSE-636 and ovarian cancer cells (UWB1), compared to the control cells without PAF treatment (as 100%). (**C**) PAFR-inhibitor, specific PAFR antibody and different concentration of antagonist ginkgolide B (1, 5, 10, 50, 100 μM) significantly (*p *< 0.05) blocked the PAF-induced proliferation in three *BRCA1*-mutant ovarian epithelial cell lines, compared to the control cells treated either with equal volume of DMSO or with IgG (as 100%). (**D, E**) Anti-apoptotic activity was significantly (p < 0.05) induced by PAF treatment in *BRCA1*-mutant at-risk HOSE-642 cells. (**F, G**) Apoptosis was significantly induced by PAF (100 nM) in wild-type HOSE-E6E7 cells, compared to the controls with 72 h treatment of equal volume DMSO. Experiments were performed at least three times (*p *< 0.05).

To investigate whether nano mole concentration of PAF, which mimics endogenous chronic inflammation, induces a significant biological effect on cell proliferation and apoptosis, three *BRCA1-*mutant ovarian surface epithelial cell lines were used. Two of them were PAFR-positive normal ovarian epithelial cell lines (HOSE-642 and HOSE-636) and one was ovarian cancer cell line (UWB1) with PAFR over expression. In addition, two normal ovarian surface epithelial cell lines (HOSE-E6E7 and HOSE-27) BRCA1-wild type and without PAFR-over expression, were used as controls. We found that after three days of incubation with different concentrations of PAF, there was a significant, as much as a 29% decrease in cell proliferation in HOSE-27 (p < 0.001) and HOSE-E6E7 cells (p < 0.05), compared with that of controls (as 100%, Fig. 5A). However, two non-cancerous *BRCA1*-mutant ovarian epithelial cells (HOSE-642 and HOSE-636) along with *BRCA1*-mutant ovarian cancer cells (UWB1) revealed a slight and significant increases in cell proliferation (Fig. 5A) at 1, 10 and 100 nM PAF (p < 0.05). After 72 h treatment with 1.0 nM PAF, cell proliferation was significantly (*p *< 0.05) decreased in the wild type HOSE cells (HOSE-27 and HOSE-E6E7). However, all three *BRAC1*-mutant ovarian epithelial cells showed no significant decrease in cell proliferation (Fig. 5B), but with slightly and significant increases. To evaluate whether PAF induced cell proliferation in *BRCA1-*mutant ovarian HOSE cells is associated with PAFR expression, cells were pretreated with PAFR inhibitor CV3988 (10 μM), PAFR antibody (1:100) and different concentrations of PAFR antagonist GB (1, 5, 10, 50, and 100 μM) for three hours followed by PAF treatment (100 nM) for 72 h. We found that the cell proliferation of all three *BRCA1-*mutant cells lines were significantly decreased (p < 0.005), especially by PAFR inhibitor and PAFR antagonist GB, compared to that of controls with equal volume of DMSO treatment (Fig. 5C). Different concentration of GB (1-100 μM) showed the similar efficacy to block PAF-induced cell proliferation, which suggests that 1 μM GB may able to reach the maximum blocking on PAF-PAFR interaction.

In addition, we examined whether cell cycle and apoptosis are differentially affected by PAF treatment in *BRCA1*-mutant and wild type ovarian surface epithelial cells. Interestingly, we found that the cell cycle analysis showed no significant changes in DNA content of G2 phase. PAF (100 nM) treatment significantly induced anti-apoptotic effects from 38.9% to 25.2%, in *BRCA1*-mutant HOSE-642 cells (Fig. 5DE). As we expected, after treatment of PAF (100 nM) for 72 hours, cell cycle analysis showed that DNA content of S phase was marginally increased from 17.02(± 0.21) % to 21.8(± 4.3)% (*p *= 0.058), and G2 phase was significantly decreased from 14% (± 4.0) to 6.0% (± 1.9) (p = 0.026) in HOSE-E6E7 cells. The apoptotic cell population was significantly increased from 16.9% (± 1.5) to 70.26% (± 2.4) (*p *= 0.001) in wild type ovarian surface epithelial cells (Fig. 5FG) when compared to the control cells treated with equivalent DMSO.

### PAF induced activation of PAFR-FAK-STAT1 networking pathways in BRCA1-mutant ovarian epithelial cells

**Figure 6:**
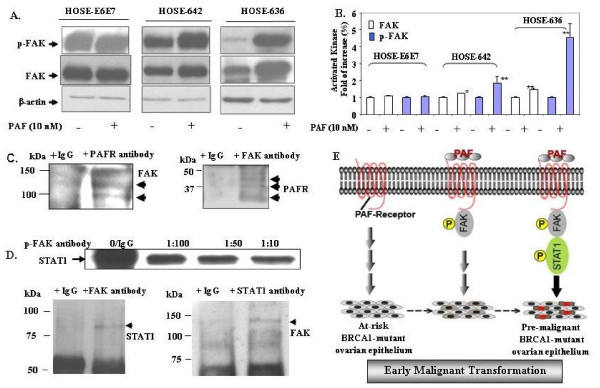
**Activation of phosphor-FAK and STAT1 is associated with PAFR in BRCA1 mutant ovarian epithelial cells**. (**A**) The specific phosphorylation of FAK in *BRCA1*-mutant at-risk HOSE-642 and HOSE-636 cells but not in wild type HOSE-E6E7 cells. **(B) **Summary of total FAK protein and phosphor-FAK protein induced by PAF treatment in wild type and BRCA1-mutant ovarian epithelial cells. The significant increases (*p < 0.05, **p < 0.01) were compared to the control cells treated with equal volume of DMSO (as 100%). (**C**) Protein-protein interaction between PAFR and FAK was detected by co-immune precipitation. Antibodies against PAFR and FAK were used for immune precipitation and incubated with protein lysate of HOSE-642 cells with 10 nm PAF treatment, and non-specific IgG was used as negative control. The immune precipitated protein complex was separated on SDS gels and detected by FAK and PAFR antibodies, respectively. (**D**). Protein-protein interaction between FAK and STAT1 was detected either by immune depletion assay or by co-immune precipitation with phosphor- and non-phospho antibodies of STAT1 and FAK. (**E**) Simplified scheme to show the integrative signal cascade and activation of PAFR, FAK, and STAT1 as early events of malignant transformation in BRCA1-mutant at-risk ovarian epithelium, particularly under the inflammatory conditions mediated lipid signaling (i.e. PAF).

Our previous studies demonstrated that PAF-PAFR is involved in the regulation of a set of protein tyrosine kinase and onco-proteins including Src and FAK in ovarian cancer cell proliferation and progression [15]. To investigate whether PAF-PAFR may play significant roles in malignant transformation in *BRCA1*-mutant HOSE cells, we have investigated both Src and FAK expression pattern in wild type HOSE-E6E7 and *BRCA1*-mutant ovarian epithelial cells (HOSE-636 and HOSE-642). There was no detectable Src protein expression in BRCA1-mutant HOSE cells by western blot (data not shown). We found that after 20 min of PAF treatment, phosphor-FAK was not changed in wild type HOSE cells, while there were significant increases of phosphorylation of FAK (p-FAK) in both *BRCA1*-mutant HOSE-642 and HOSE-636 cell lines (Fig. 6A). The total FAK protein expression was also induced by PAF treatment in HOSE-642 and HOSE-636 cells, but not in wild type HOSE-E6E7 cells (Fig. 6AB). Using co-immune precipitation, our data showed that both phosphor-FAK protein and PAFR can be cross-detected by their specific antibodies in western blots (Fig. 6C). Although we have not yet provided the detailed mechanisms of protein-protein interaction and the dynamic interactions of these protein interactions, our preliminary data suggest that upon PAF treatment and binding to PAFR, the PAF-mediated inflammation signal could be transduced from extracellular domain of PAFR to FAK through protein-protein interaction in *BRCA1*-mutant HOSE-642 cells. A similar co-immune precipitation approach demonstrated that there are significant cross-immune reactions and protein-protein interactions between FAK and STAT1, either by p-FAK antibody immune depletion or by co-immune precipitation (Fig. 6D). While this observation may require further validation in other BRCA1-mutant epithelial cells, our data together with the previous findings of FAK-STAT1 interaction in cell migration, adhesion through protein-protein interaction and auto-phosphorylation [33,34] strongly suggest that the inflammatory mediator PAF plays a significant role in inducing malignant transformation of the at-risk epithelium through activation of multiple targets and networking cascade of PAFR, FAK and STAT1 in coordination with *BRCA1*-dysfunction in ovarian epithelial cells (Fig. 6E).

## Discussion

*BRCA1 *is a tumor-suppressor gene in which germ-line mutations lead to a predisposition of breast and ovarian cancer [35]. Normal BRCA1 regulates multiple nuclear processes including DNA repair and recombination, checkpoint control of the cell cycle [36]. There is growing interest in the identification of the early events and molecular signals involved in the predisposition to cancer associated with BRCA mutations [11]. The present study provides the first evidence to demonstrate that *BRCA1*-mutation related cancer risk and malignant transition may be associated with accumulation of potent pro-inflammatory lipid PAF and over expression PAFR in non-malignant ovarian epithelial cells. Over-expression of PAFR has been observed in malignant melanoma [37] and ovarian cancer [15]. We have found that PAFR is also over expressed in non-cancerous *BRCA1*-mutant epithelial cells of the ovarian surface and distal fallopian tube, compared to the wild type normal HOSE cells and fallopian tube surface epithelial cells. This observation may require additional confirmation with a larger set of non-malignant *BRCA1*-mutant cells and tissue specimens. However, our study provides clear evidence that potent pro-inflammatory mediators such as phospholipid PAF and its receptor PAFR play significant roles in inducing anti-apoptosis and malignant potential of the at-risk epithelial cells in coordination with BRCA1 dysfunction (Fig. 5 and 6).

In addition, we found that cell proliferation was increased ~20% in all *BRCA1*-mutant HOSE cell lines by PAF treatment. This was less than the induced effect observed in many ovarian cancer cell lines (>100% increase) [15], but significantly decreased cell proliferation in normal wild type HOSE cell lines. Moreover, PAFR inhibitors blocked the PAF-induced cell proliferation and p-STAT expression in all *BRCA1*-mutant cell lines. This suggests that over expression of PAFR could be an essential sensor for the *BRCA1*-mutant surface epithelial cells to probe potent lipid inflammatory mediators found in the micro-environment, including the conditions of incessant ovulation cycles [38,39]. Correspondingly, PAF-induced differential effects in cell proliferation, cell cycle and apoptosis in the wild type and non-malignant *BRCA1*-mutant HOSE cells provide additional confirmation that BRCA1-mutation and dysfunction is associated with the phenotypic PAF response, especially under inflammatory conditions through the PAFR pathway (Fig. 5). These combined findings suggest that dysfunction of BRCA1 and over expression of PAFR may contribute and account for the greater likelihood of malignant transformation of BRCA1-mutant at-risk epithelial cells, compared to wild type ovarian or tubal epithelium.

Signal transducers and activators of transcription (STATs) are a family of cytoplasmic proteins that function as signal messengers and transcription factors involved in cellular responses to cytokines and growth factors [40]. A line of evidence supports that STAT pathways play a significant role in malignant transformation [8,41]. For example, a recent report showed that STAT1 phosphorylation determines Ras oncogenicity through p27 protein [42]. Activated STAT1, STAT3 and STAT5 are often observed in solid and liquid tumors, and are required for tumor transformation and progression which involves a set of oncogenes such as EGFR, Ras, Src, and FAK [8,43-45]. Here we found that phosphorylation of STAT1, as well as STAT4 and STAT6 (not shown) were simultaneously activated by PAF treatment in *BRCA1-*mutant ovarian cancer cells (UWB1). Activated STAT1 phosphorylation was further confirmed in two additional *BRCA1-*mutant HOSE cell lines, but not shown in wild type HOSE-E6E7 and ovarian cancer (OVCA429) cells (Fig. 4). In addition, PAF-induced STAT1 phophorylation was partially blocked by PAFR inhibitors in *BRCA1-*mutant HOSE-642 and UWB1 cells. This suggests that PAFR is involved in STAT1 activation in *BRCA1-*mutant HOSE cells. In the BRCA1-wild type cells, functional BRCA1, as a tumor suppressor, acts in concert with STAT1 to activate transcription of a subset of IFN-γ gene targets and growth inhibition by cytokine [46]. Here we provide independent evidence that coordination of abnormal function of BRCA1 and activation STAT1 phosphorylation could induce HOSE cell proliferation and anti-apoptosis in *BRCA1-*mutant cells, particularly under inflammatory conditions.

Focal Adhesion Kinase (FAK) is a key mediator of signaling induced by integrins that play an instrumental role in many cellular functions including cell survival, proliferation [47] and stem cell signaling [48]. FAK is often associated with cancer transformation [44], progression, and metastasis [15] through the site specific phosphor-activation. Our findings are consistent with many other observations that FAK directly interacts with STAT1 [33,34], through phosphorylation of FAK to regulate the cellular functions such as cell apoptosis, migration, invasion and metastasis [49-51]. Although further investigation is required to confirm this finding in different BRCA1-mutant cell lines, our data suggests that FAK may have distinct activation pathways in non-malignant *BRCA1*-mutant epithelial cells and in wild type malignant progressive cells [51]. Nevertheless, the evidence for the assignation of the roles of inflammatory lipids, including PAF through paracrine regulation in cancer initiation, particularly in BRCA-mutant epithelial cells is likely as one of key mechanisms, but require further investigation. It may be restricted to specific situations, depending on mutation sites, tissue type and microenvironment. Here we provided a unique BRCA1-mutant at-risk *in vitro *model and demonstrated an evidence that coordinated activation of PAF-PAFR, and FAK and STAT pathways, with the abnormal BRCA1 functions may contribute to the significant increases of malignant potential and early events of tumor transformation in at-risk ovarian epithelium (Fig 6E). This *in vitro *at-risk ovarian epithelium may represent a valuable model to understand the early molecular events of malignant transformation and development. Certainly, it requires further molecular approaches including networking pathway and target knock-down *in vitro *and *in vivo *models to confirm that the signal axis of PAF/PAFR-FAK-STAT pathway is significantly mediated in the early events of ovarian epithelial malignant development.

## Conclusions

Our findings showed that PAFR was over expressed in BRCA1-mutant ovarian and fallopian tubal epithelial cells, PAF specifically induced phosphorylation of FAK and STAT1 in BRCA1-mutant ovarian epithelial cells, and associated with protein-protein interaction between PAFR and FAK and FAK and STAT1. These findings strongly suggest that potent inflammatory lipid mediators such as PAF and its receptor (PAFR) are not only involved in cancer progression and metastasis, but they are also significantly involved in early malignant transformation through phosphor-FAK/STAT1 networking and anti-apoptosis pathway in BRCA1-mutant dysfunctional at-risk ovarian epithelium.

## List of abbreviations

PAF: Platelet activating factor; PAFR: Platelet activating factor receptor; LPA, HOSE: Human Ovarian surface epithelial cells; UWB1: ovarian cancer cell line UWB1.289; MTT: 3-(4,5-dimethylthiazol-2-yl)-2,5-diphenyltetrazolium bromide; GB: ginkoglide B; FAK: Focal adhesion kinase; STAT1: Signal Transducers and Activators of Transcription family protein 1.

## Competing interests

The authors declare that they have no competing interests.

## Authors' contributions

LZ, DW, JW, DE, LMB, JR, LJ, VS carried out the experiments, protocol design, data analysis and interpretation of data and drafting the manuscript. WQ, AV, CC, DWC, JW, SCM, and BY are involved in experiment design, acquisition, interpretation and manuscript preparation. All authors read and approved the final manuscript.

## Author details

1 Department of Obstetrics and Gynecology, Brigham and Women's Hospital, Boston, MA, USA

2 Channing Laboratory, Brigham and Women's Hospital, Boston, MA, USA

3 Obstetrics and Gynecology Department, Peking University People's Hospital, Beijing, China

4 Department of Gynecologic Oncology, University of Texas M. D. Anderson Cancer Center,

Houston, TX, USA

5 Department Pathology, Brigham and Women's Hospital, Boston, MA, USA
